# An indirect enzyme-linked immunosorbent assay for rapid and quantitative assessment of Type III virulence phenotypes of *Pseudomonas aeruginosa *isolates

**DOI:** 10.1186/1476-0711-4-22

**Published:** 2005-12-23

**Authors:** Li Li, Michel Ledizet, Kalipada Kar, Raymond A Koski, Barbara I Kazmierczak

**Affiliations:** 1 Department of Internal Medicine (Infectious Diseases), Yale University School of Medicine, New Haven CT, USA; 2 Section of Microbial Pathogenesis, Yale University School of Medicine, New Haven CT, USA; 3 L^2^Diagnostics, LLC, New Haven CT, USA

## Abstract

**Background:**

The presence of a Type III secretion system in clinical isolates of *Pseudomonas aeruginosa *is associated with severe disease and poor outcomes in infections caused by this pathogen. We describe an indirect enzyme-linked immunosorbent assay that rapidly and quantitatively detects two exotoxins, ExoU and ExoT, and two structural components, PopD and PcrV, of the *P. aeruginosa *Type III secretion system after in-vitro growth in a calcium-free minimal medium.

**Methods:**

We used this assay to characterize the Type III secretion phenotype of 74 clinical isolates of *P. aeruginosa*. Findings were compared with results of standard immunoblotting and correlated with Type III secretion-dependent virulence of isolates toward cultured epithelial cells.

**Results:**

Results of the ELISA assay were concordant with immunoblot detection of the secreted antigens for 73 of 74 isolates. The Type III secretion phenotype assessed by this immunoassay predicted bacterial virulence toward epithelial cells in vitro for all but five of the clinical isolates.

**Conclusion:**

The availability of an ELISA assay for rapid detection of Type III secreted virulence factors will facilitate large clinical studies to examine whether the Type III secretion phenotype of a *P. aeruginosa *isolate predicts the course of clinical disease in a patient and should be taken into account in determining optimal treatment strategies for infected patients.

## Background

*Pseudomonas aeruginosa *is a gram-negative human pathogen that causes acute infections associated with significant morbidity and mortality, particularly among hospitalized patients and immunocompromised individuals [1]. Many *P. aeruginosa *isolates express a specialized protein secretion system, called the Type III secretion system (T3SS) [2,3]. Three studies have demonstrated that more severe manifestations of *P. aeruginosa *acute infection, such as bacteremia, relapsing pneumonia, pneumonia plus bacteremia, or death attributable to *P. aeruginosa *infection, are associated with the expression of T3SS proteins by patient clinical isolates [4-6]. These observations are in agreement with multiple in vitro and animal studies which have demonstrated that the T3SS is required for both cytotoxicity toward tissue culture cells and virulence in animals following *P. aeruginosa *infection [7,8].

The T3SS of *P. aeruginosa *consists of a multiprotein channel that spans the inner membrane, periplasm and outer membrane of the bacterium (reviewed in [9,10]). This channel mediates the secretion of four exotoxins, namely ExoS, ExoT, ExoU and ExoY [2,8,11,12]. Three additional proteins, called PopB, PopD and PcrV, are also secreted by the T3SS. In the presence of mammalian cells, PopB, PopD and PcrV enable formation of a pore structure, or "translocon", in the eukaryotic cell membrane that allows passage of the exotoxins into the cytoplasm of mammalian host cells [13,14]. Although the in vivo signals which trigger T3S are poorly understood, production and secretion of the effectors can be induced in vitro by growing the bacteria in media containing a calcium chelator such as nitrilotriacetic acid (NTA) [9].

The individual *P. aeruginosa *exotoxins have been characterized in significant detail. ExoU is a phospholipase which causes cell necrosis; it requires an as-yet-unidentified eukaryotic co-factor for activity [15-18]. Expression of ExoU by *P. aeruginosa *strains strongly predicts virulence in murine models of acute pneumonia [7]. There is a trend toward more severe disease in patients with ventilator-associated pneumonia infected with ExoU-secreting isolates in the study of Hauser *et al*. which is not statistically significant (*p *= 0.22), but the relationship is stronger than that measured for any other effector [5]. The effectors ExoT and ExoS are highly related bifunctional proteins with both GTPase activating protein (GAP) activity toward Rho-family proteins, and ADP-ribosyltransferase activity toward non-overlapping sets of substrates [19-23]. Both proteins inhibit actin cytoskeleton rearrangements, resulting in tissue culture cell detachment and inhibition of phagocytosis. Of the two, ExoS contributes more to *P. aeruginosa *virulence in animal models, in a manner dependent upon its ADP-ribosyltransferase activity [24,25]. The last known effector, ExoY, is a calmodulin-dependent adenylate cyclase [12]. No clear role for ExoY has been established in *P. aeruginosa *pathogenesis [25,26]. Clinical *P. aeruginosa *isolates express various subsets of these four effectors, which has led many investigators to hypothesize that the varied manifestations of *P. aeruginosa *infection may in part reflect the exotoxin complement produced by the infecting organism.

The association of T3S and disease severity suggests that knowing the T3SS phenotype of a clinical isolate may aid physicians involved in treating patients from whom *P. aeruginosa *is cultured. The isolation of an ExoU producing strain may prompt a more aggressive treatment algorithm or longer duration of therapy, while the isolation of a T3SS-negative isolate may support less aggressive management in select patient populations. Several investigators are developing treatment strategies that specifically target the T3SS, such as antibodies directed against PcrV or small molecule inhibitors of the T3SS itself [13,27-31]. To use such strategies most effectively, a treating physician would require timely information about the capacity of a clinical isolate to express the T3SS and the specific exotoxins that it secretes. Unfortunately, the immunoblotting techniques used by research laboratories to characterize T3SS profiles of *P. aeruginosa *isolates are ill-suited to either large-scale clinical trials or to rapid clinical sample testing. We have therefore developed a rapid quantitative test which detects four proteins secreted by the T3SS, namely PcrV, PopD, and the two exotoxins ExoU and ExoT. Unlike immunoblotting, this assay can be performed on hundreds of samples per day in a format accessible to most clinical microbiology laboratories. We show that our assay results are concordant with those obtained by immunoblotting and accurately predict virulence of clinical isolates toward epithelial cells. We suggest that this assay may be ideally suited for large-scale clinical studies designed to examine prospectively the impact of T3SS-related virulence factors on *P. aeruginosa *disease in human patients.

## Methods

### Bacterial strains and culture media

Reference bacterial strains are listed in Table 1. Bacteria were maintained as frozen stocks in 15% glycerol at -80°C and freshly streaked to Luria Broth (LB) agar plates prior to each experiment. For analysis of Type III secretion, a single colony was used to inoculate 1–3 ml of MinS, which contains the divalent cation chelator NTA, [32] and grown with vigorous aeration at 37°C.

**Table 1:** Reference bacterial strains used in this study

Strain	Relevant characteristic(s)^*a*^	Reference(s) or source
PA103	Virulent lung isolate of *P. aeruginosa*; known type III secreted effector proteins are ExoT and ExoU	[45, 46]
PAO1	Reference *P. aeruginosa *strain; known type III secreted effector proteins are ExoS, ExoT and ExoY	[47, 48]
mutN	PA103*pscJ*::Tn5Gm^R^; defective in Type III secretion	[39]

PA103ΔU	PA103 with an in-frame deletion of amino acids (aa) 330–571 of ExoU	[35]
PA103ΔT	PA103 with a *xylE aacC1 *cassette replacing aa 36–348 of *exoT*; Gm^R^	[35]
PA103ΔUΔT	PA103ΔU with a *xylE aacC1 *cassette replacing aa 36–348 of *exoT*; Gm^R^	[35]

PA103Δ*pcrV*	PA103 with an in-frame deletion of aa 10–277 of PcrV	(L. Li, L. Mijares, and B. I. Kazmierczak, submitted for publication)

PA103Δ*popD*	PA103 with an in-frame deletion of aa 7–274 of PopD	(L. Li, L. Mijares, and B. I. Kazmierczak, submitted for publication)

PA103Δ*popB*	PA103 with an in-frame deletion of aa 11–371 of PopB	(L. Li, L. Mijares, and B. I. Kazmierczak, submitted for publication)

^*a *^Gm^R^, gentamicin-resistant.

### *Pseudomonas aeruginosa *clinical isolates

Seventy-four clinical isolates of *Pseudomonas aeruginosa *were obtained from the Laboratory of Clinical Microbiology at Yale-New Haven Hospital during a 6 month period. Clinical isolates were identified as *P. aeruginosa *if they grew as non-lactose fermenting colonies on MacConkey agar, were oxidase-positive, could utilize acetamide as a sole carbon source, and gave a characteristic zone of inhibition to colistin on Kirby-Bauer plates. The presence of characteristic pigment and "grape-like" odor were also noted. Any isolates which could not be conclusively identified based on the above criteria were also tested for the ability to grow in broth at 42°C. The date of isolation and the type of clinical specimen from which the strain was obtained were recorded. Each strain was given a unique identifying number between CI001 and CI079. No patient identifiers were recorded, and no clinical information was available to differentiate isolates from acutely infected patients versus isolates from Cystic Fibrosis patients. The most common specimen types were blood (n = 19), urine (n = 19), sputum (n = 18); the remainder of the isolates derived from deep wound, bone, sinus, bile, eye or stool cultures. Each strain was cultured in 3 ml LB medium and stored as a frozen stock in 15% glycerol.

### Purification of ExoU and PcrV

Native ExoU and PcrV were purified from *Pseudomonas *culture supernatants. *Pseudomonas aeruginosa *strain PA103 was grown thirteen hours in MinS. Culture supernatant was pooled and concentrated by ultrafiltration on a Pellicon XL cassette with a Biomax-10 membrane (Millipore, Billerica MA). The buffer was changed to 25 mM TrisHCl pH 8 by dilution and concentration on the same device. The material was then applied to a MonoQ HR5/5 ion-exchange column (Amersham Pharmacia) and eluted with a 0–0.6 M NaCl gradient in 25 mM TrisHCl pH8. This procedure yielded ExoU and PcrV with purities estimated at ≥90%. ExoU, which is the most abundant protein in culture supernatants, was identified by its apparent molecular weight. As PopD and PcrV have similar apparent molecular weights, the identity of PcrV was determined by tryptic digestion of the purified protein extracted from a gel slice following SDS-PAGE. The tryptic fragments were analyzed by MALDI-MS (Keck Biotechnology Center, Yale University). An automated search of several databases unambiguously confirmed that the purified protein was PcrV.

### Hybridomas producing monoclonal antibodies against ExoU and PcrV

All animal work was performed according to protocols approved by the Yale University Institutional Animal Care and Use Committee (IACUC). Six-week old female Balb/c mice (NCI) were immunized with 20 μg of ExoU or 5 μg of PcrV in complete Freund's adjuvant. The mice were boosted twice at 3 week intervals with the same amount of antigen in incomplete Freund's adjuvant. Two weeks after the second boost, spleen cells were harvested and fused with Ag8 myeloma cells (ATCC). The resulting hybridoma cells were selected in hypoxanthine, aminopterin, and thymidine (HAT) culture medium using standard procedures. Hybridoma cells underwent three rounds of cloning by limiting dilution and were then expanded and frozen.

After each round of cloning, positive cultures were identified by an enzyme-linked immunoassay using purified ExoU or PcrV as antigen. Bound antibodies were detected with an alkaline phosphatase-conjugated goat anti-mouse IgG antibody (Sigma, St Louis MO). The chromogenic substrate paranitrophenyl phosphate (PNPP) (Sigma) was used in all experiments.

### Hybridomas producing monoclonal antibodies against ExoT and PopD

We were unable to purify soluble ExoT and PopD from culture supernatants. We therefore used insoluble virulence factor aggregates, which form after prolonged culture in calcium-free medium, as a source of antigen [8]. These aggregates were collected from 18 hour MinS cultures of strain PA103, washed several times with phosphate buffered saline (PBS) and solubilized with 4% SDS.

Six-week old female Balb/c mice were immunized with 100 μg of solubilized aggregates emulsified with complete Freund's adjuvant. Mice were boosted twice at three week intervals with the same amount of antigen in incomplete Freund's adjuvant. Hybridomas were purified as described above. Antibody-producing hybridomas were identified by ELISA, using supernatants from overnight cultures of *P. aeruginosa *strain PAO1 as antigen. (PAO1 does not produce ExoU, which avoided the isolation of additional anti-ExoU hybridomas.) Bound antibodies were detected as described above. The specificity of the antibodies produced was determined in two steps. The molecular weight of the antigen recognized was determined by immunoblotting against a PAO1 culture supernatant (as described below). The identity of the antigen recognized was then confirmed by preparing culture supernatants from mutant laboratory strains lacking individual T3SS components (Table 1). Antibody binding to these culture supernatants was determined in immunoblotting and ELISA experiments.

### Purification and characterization of monoclonal antibodies

Hybridomas were grown in Hybridoma-SFM serum-free medium (Invitrogen, Carlsbad CA) after a short adaptation period. Each monoclonal antibody was purified from hybridoma culture supernatant by affinity chromatography on protein G-agarose (Amersham, Piscataway NJ). Isotypes were identified with the ImmunoPure Monoclonal Antibody Isotyping Kit (Pierce, Rockford IL).

We produced several monoclonal antibodies specific for each of the four T3SS proteins under investigation. To determine whether more than one epitope was recognized in each protein, we performed antibody competition assays in an ELISA format. Each purified immunoglobulin was covalently labeled with horseradish peroxidase (HRP) using EZLink Peroxidase (Pierce) according to the manufacturer's instructions. 96-well plates were coated with a PA103 culture supernatant diluted in carbonate buffer. Each antibody (0.5 – 8 μg/ml) was allowed to react with the bacterial proteins in the presence of a 50-fold excess of unlabeled competing antibody. HRP conjugated antibodies were detected with SureBlue™ chromogenic substrate (KPL, Gaithersburg MD). The unlabeled antibody was considered to bind to the same epitope as the labeled antibody if it reduced binding of the labeled antibody by at least 75%. We found that the reactivity of IgG_1 _antibodies was greatly diminished by the HRP conjugation. As an alternative, we therefore non-covalently labeled each IgG_1 _antibody with an alkaline phosphatase-conjugated anti-IgG_1 _Fab fragment (Zenon™ technology, Invitrogen). The competition assay was performed as before, except that alkaline phosphatase labeled antibodies were detected with PNPP. Identical conclusions were reached with the two labeling methods.

### Polyclonal antiserum against secreted T3SS components

Insoluble aggregates containing T3SS components were prepared from PA103 supernatants as described above and dissolved in 4% SDS. Two New Zealand white rabbits (Cocalico Biologicals, Inc.) were each immunized with 100 μg of solubilized antigen emulsified in complete Freund's adjuvant. Three weeks later the rabbits received a booster injection of 100 μg of antigen in incomplete Freund's adjuvant. Serum samples were collected at two-week intervals for four months. The endpoint titer of this antiserum was >1:32,000 by ELISA with PA103 culture supernatant used as antigen.

### Detection of Type III secretion system components by immunoblotting

T3SS components were detected by immunoblotting using our rabbit polyclonal antiserum or monoclonal antibodies. Proteins in 1 mL samples of *Pseudomonas *culture supernatant were concentrated by precipitation with 10%(v/v) trichloroacetic acid (TCA). The pellets were washed once with acetone and redissolved in SDS gel electrophoresis sample buffer. The proteins were separated by SDS-PAGE and transferred to PVDF (Immobilon-P, Millipore). Blots were blocked in Tris-buffered saline plus 0.1% Tween-20 (TBST) containing 5%(w/v) dry milk for 30 min prior to applying anti-T3SS rabbit antiserum at a dilution of 1:20,000 in TBST plus milk. HRP-conjugated goat anti-rabbit IgG (Biorad) diluted 1:2,000 was used as secondary antibody. Proteins were visualized by incubating membranes in 225 μM coumaric acid (Sigma), 1.25 mM 3-aminophthalhydrazide (Fluka) and 0.009% hydrogen peroxide (Fisher Scientific) in 100 mM Tris-HCl pH 8.5 for 1 min. Chemiluminescence was detected with an Image Station 2000R using 1D Image Analysis software v. 3.6 (Kodak). The exotoxins ExoU, ExoT and ExoS as well as the translocon components PopB and PopD could be detected simultaneously. The identity of each protein band was confirmed by comparing the immunoblot pattern obtained from culture supernatants of wild-type strains versus isogenic defined mutant strains (Table 1). PcrV was not detected by this polyclonal serum, but could be detected by a similar procedure with the monoclonal antibody RC (this study) used at a concentration of 0.1 μg/ml, followed by incubation with HRP-conjugated goat anti-mouse IgG (Biorad).

### Quantitative detection of components of the Type III secretion system by ELISA

Each *Pseudomonas *clinical isolate was tested for production of T3SS components in 2–6 independent assays using an ELISA optimized by multiple checkerboard titrations. Each *Pseudomonas *strain was grown with vigorous agitation for 12–16 hours in 1–3 ml MinS at 37°C. Culture supernatants were cleared by centrifugation at 7,000 × *g *for ten minutes, then diluted 20-fold with 50 mM carbonate buffer pH 9.6 and used to coat 96-well plates for two hours at 37°C. Monoclonal antibodies were diluted to 1 μg/ml in PBS containing 0.05% Tween-20 (PBS-T) and 0.5% dry milk, applied to the plates, and allowed to react for one hour at room temperature. A secondary antibody (alkaline phosphatase-conjugated goat anti-mouse IgG, Sigma) was applied at a dilution of 1:3,000 in PBS-T and allowed to react for one hour at room temperature. After both incubations, unbound antibody was removed by washing three times with PBS-T. Bound antibody was detected with PNPP.

Negative control wells were coated with a culture supernatant from the *Pseudomonas *mutN strain, which does not secrete any component of the T3SS. For each monoclonal antibody, the optical density observed in the negative control wells was subtracted from all experimental values. On every 96-well plate, eight positive control wells were coated with a frozen reference aliquot of PA103 culture supernatant. The signals observed with experimental clinical isolates were expressed as a percentage of the signal obtained with the PA103 culture supernatant. Two monoclonal antibodies were used to detect each T3SS component, and the signals obtained with each were averaged. A strain was considered positive for a T3SS component if the averaged antibody signal was at least 10% that seen with the PA103 strain.

### Detection of genes encoding ExoS, ExoT, ExoU, PcrV, and PopD by PCR

The presence of genes encoding T3SS components was determined by PCR amplification of bacterial DNA using pairs of gene-specific primers. Single colonies were suspended in 10 μl of water and boiled for five minutes. One microliter of this preparation was used as the source of DNA for PCR reactions. Table 2 lists the primers used as well as the cycling conditions.

**Table 2:** PCR parameters for the detection of genes encoding TTSS components

Gene	Forward & reverse primer sequences (all primers listed 5' to 3')	PCR conditions
*exoS*	S1: ATGGCGTGTTCCGAGTCA	Annealing temperature step-down 1°C/cycle (8 cycles) from 62°C to 55°C, followed by 25 cycles at 54°C. All extensions carried out at 72°C for 90 sec.
	S2: AGGTGTCGGTTCGTGACGTCT	
*exoT*	T1: GAGAGGCTGGCGAAGGATCAC	
	T2: CCAGGTCCAGAGCATCGAGCA	
*exoU*	U1: GGCACATATCTCCGGTTCCTTC	
	U2: TCAACTCAGCTGCCAACCATGC	

*pcrV*	V1: ATGGAAGTCAGAAACCTTAATGCCG	Annealing temperature step-down 1°C/cycle (8 cycles) from 58°C to 51°C, followed by 25 cycles at 50°C. All extensions carried out at 72°C for 75 sec.
	V2: CTAGATCGCGCTGAGAATGTC	
*popD*	D1: ATGATCGACACGCAATATTCCC	
	D2: TCAGACCACTCCGGCCGCCGCA	

### Cytotoxicity assay

The ability of *Pseudomonas *strains to induce necrosis of HeLa cells (ATCC) was determined by measuring lactate dehydrogenase (LDH) release, as described before [33]. Each clinical isolate was assayed in triplicate in 2–3 independent assays. A strain was scored as positive in this assay if the bacteria caused at least 25% as much necrosis as the laboratory strain PA103 at 2 h post-infection.

### Cell rounding assay

*Pseudomonas *bacteria were grown as for cytotoxicity assays prior to infecting HeLa cells at an MOI of ca. 10. Each strain was assayed in triplicate in 2–3 independent assays, with PAO1 and mutN strains serving as positive and negative controls for each experiment. Cells were observed by phase-contrast microscopy after three hours of bacteria-host cell contact. A strain was considered to cause cell-rounding if fewer than 50% of HeLa cells remained attached and maintained their characteristic elongated cell shape at 3 h post-infection.

## Results

### Monoclonal antibodies against ExoU, ExoT, PcrV, and PopD

**Figure 1:**
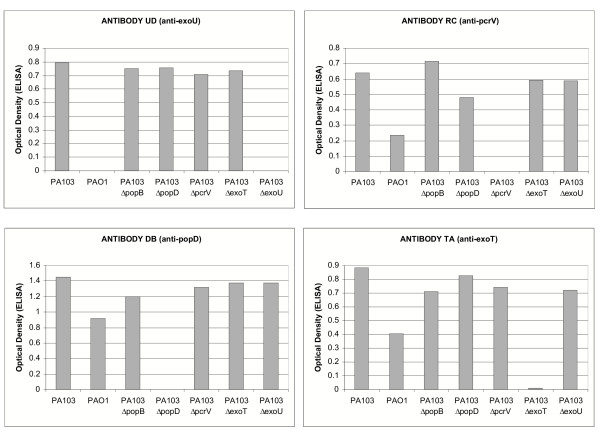
**Specificity of monoclonal antibodies against TTSS components. **Monoclonal antibodies generated against ExoU, PcrV, PopD and ExoT were tested in the ELISA assay format against culture supernatants prepared from PAO1, PA103 and defined mutants isogenic with the laboratory strain PA103. Note that PAO1 does not produce or secrete ExoU.

We generated twelve novel hybridoma cell lines producing monoclonal antibodies specific for T3SS components (Table 3). The specificity of each antibody in our ELISA assay was verified by testing its reactivity against culture supernatants of isogenic strains lacking specific T3SS components. These experiments showed that each monoclonal antibody is specific for a single T3SS component (Figure 1 and data not shown). The specificity of each antibody was further confirmed by immunoblotting experiments (data not shown). Of note, none of the anti-ExoT monoclonal antibodies recognized the closely related protein ExoS in immunoblots. Each monoclonal antibody was purified and its isotype determined. As Table 3 shows, most antibodies consisted of γ1 heavy chains and κ light chains.

**Table 3:** Isotypes of monoclonal antibodies recognizing TTSS components ExoT, ExoU, PcrV, and PopD

TTSS Component Recognized	Monoclonal Antibody	Isotype – Heavy Chain	Isotype – Light Chain
ExoT	TA	IgG_1_	Kappa
	TB	IgG_1_	Kappa
	TC	IgG_1_	Kappa

ExoU	UA	IgG_1_	Lambda
	UB	IgG_1_	Kappa
	UC	IgG_1_	Kappa
	UD	IgG_2a_	Kappa

PcrV	RA	IgG_1_	Kappa
	RC	IgG_1_	Kappa
	RD	IgG_1_	Kappa

PopD	DA	IgG_2b_	Kappa
	DB	IgG_2a_	Kappa

**Figure 2:**
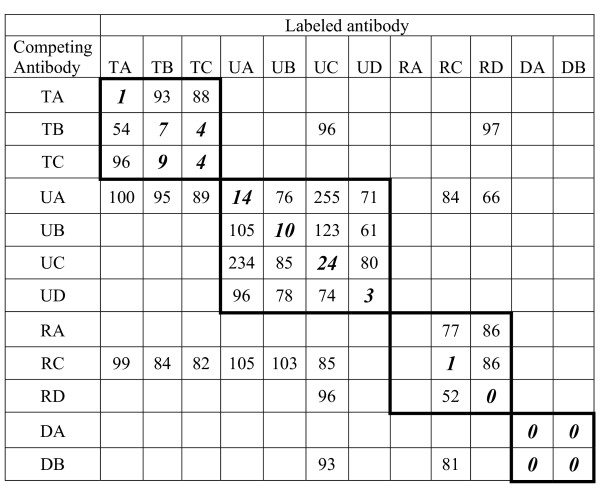
**Competition between monoclonal antibodies against TTSS. **Binding of antibodies to antigens derived from PA103 culture supernatants was measured in an ELISA assay as described in Material and Methods. Numbers show the percentage of labeled antibody binding in the presence of a 50-fold excess of competing antibody. Inhibition of 75% or more (numbers in bold italics) indicates that the two antibodies compete for the same epitope. The binding of the RA antibody was too weak to yield reliable quantitative results.

ExoU, ExoT, PcrV, and PopD were each recognized by several monoclonal antibodies. In order to determine whether the antibodies recognized the same epitopes, we performed antibody competition experiments. Representative results are shown in Figure 2. The antibodies recognize four distinct epitopes on ExoU; two of these anti-ExoU antibodies (UA and UC) exhibit cooperative binding. RC and RD antibodies recognize distinct epitopes on PcrV, while DA and DB recognize overlapping or identical epitopes on PopD. One epitope of ExoT is recognized by TA while another is recognized by both TB and TC.

For all further experiments, we selected a panel of monoclonal antibodies (TA, TB, UA, UB, RC, RD, DA, and DB) based upon their specificity and sensitivity.

### Quantitative detection of T3SS components by ELISA

**Figure 3:**
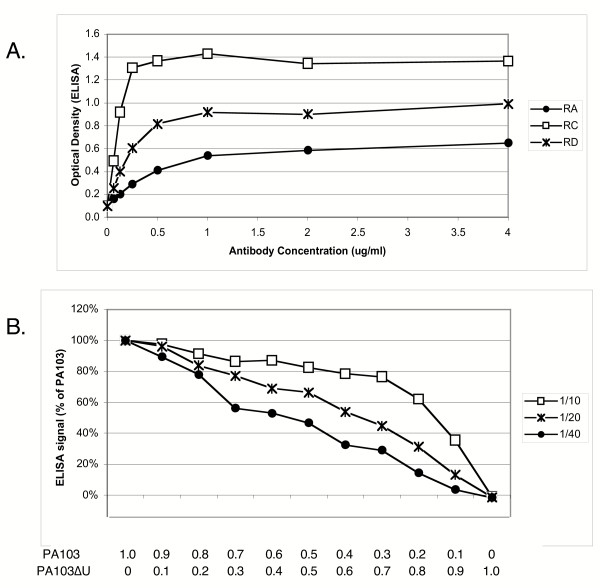
**Optimization of ELISA assay parameters. **A. Increasing concentrations of monoclonal antibodies RA, RC and RD were used in the ELISA assay with a constant amount of PA103 culture supernatant serving as bound antigen. B. PA103 and PA103ΔU culture supernatants were mixed in the proportions indicated to vary the amount of ExoU present in each sample while holding other secreted antigens constant. The mixed supernatants were diluted with carbonate buffer as indicated, then assayed by ELISA using antibodies UA and UB. Values represent the average of the signal obtained with the two antibodies.

ELISA assay parameters were optimized in a series of preliminary experiments. Each antibody was evaluated at several concentrations ranging from 0.05 to 9 μg/ml, using PA103 culture supernatant as antigen. Panel A of Figure 3 shows the results of a titration experiment for the three monoclonal antibodies specific for PcrV, namely RA, RB and RC. Increasing the antibody concentration led to higher optical densities. For these and all other antibodies, there was little benefit to be gained by using concentrations higher than 1 μg/ml (data not shown). We therefore performed all subsequent experiments at an antibody concentration of 1 μg/ml.

To verify that the result of the ELISA is proportional to the amount of antigen in the sample analyzed, we mixed PA103 culture supernatant with a culture supernatant derived from an isogenic defined mutant lacking a single T3SS component. These mixtures thus contain a variable amount of one T3SS component and relatively constant concentrations of other proteins. Figure 3, panel B shows the results of such an experiment with the antibodies UA and UB, which are specific for ExoU. The data shown are the average of the values obtained for the two antibodies. The samples mixed were culture supernatants from PA103 (wild-type) and PA103ΔU, an isogenic mutant lacking the *exoU *gene. When the mixed culture supernatants were diluted 40-fold with carbonate buffer prior to coating the plates, the ELISA signal was proportional to the percentage of ExoU-containing PA103 supernatant in the antigen mixture. Thus under these conditions, ELISA results are proportional to the amount of ExoU in the sample tested. Coating plates with culture supernatant mixtures diluted 10- or 20-fold increased the signal observed at low ExoU concentrations, but resulted in a flattening of the dose-response curve at higher ExoU concentrations (Fig. 3B). Similar results were obtained with ExoT, PopD and PcrV antigen-antibody combinations (data not shown). We used a 20-fold dilution of culture supernatant for our experiments, unless otherwise indicated, as this provides good sensitivity and correlation with the amount of antigen plated.

### Production and secretion of T3SS components by *Pseudomonas *clinical isolates

We studied the secretion of T3SS components by 74 *P. aeruginosa *isolates obtained from the Laboratory of Clinical Microbiology at Yale-New Haven Hospital using eight monoclonal antibodies in our ELISA format as described above. In each assay, frozen aliquots of reference PA103 and mutN culture supernatants were used as positive and negative controls, respectively. We considered a strain to be positive if it secreted at least 10% of the amount of T3SS component present in the PA103 supernatant. We arrived at this cut-off by determining the standard deviation of our negative control, the mutN supernatant, in over 20 independent assays performed by two different operators (K. K. and M. L.). Values three standard deviations above the negative control were considered positive; this corresponded to 10% of the positive control when averaged for all 8 antibodies (range, 6–17%).

**Figure 4:**
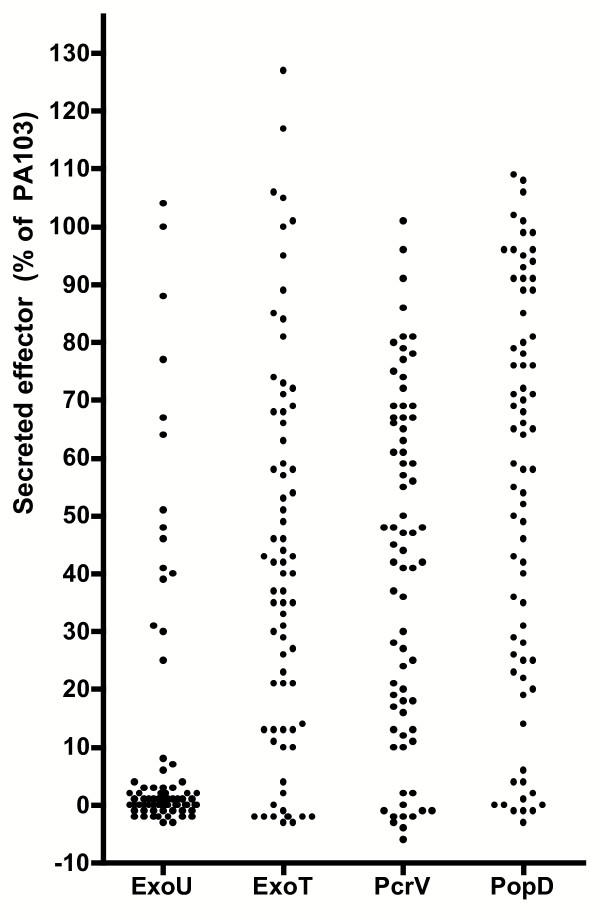
**T3SS component production by clinical isolates as measured by ELISA. **Each point represents the amount of ExoU, ExoT, PcrV or PopD secreted by one of the 74 clinical isolates as determined by ELISA. Each value is expressed as the percentage of protein present in PA103 supernatants (positive control).

Using this criterion, 15 of 74 isolates (20%) were positive for ExoU, ExoT, PcrV, and PopD (Table 4). 47 isolates (64%) were positive for ExoT, PcrV, and PopD but negative for ExoU. Finally, 12 isolates (16%) were negative for all four T3SS components by ELISA. The proportion of ExoU positive strains was particularly high among isolates derived from blood (6 out of 19, or 32%), as has been described before [4]. These results are presented graphically in Figure 4, in which each data point represents the amount of ExoU, ExoT, PcrV or PopD secreted by an individual isolate as measured by ELISA.

**Table 4:** Genotypes and phenotypes determined for 74 clinical isolates.

Genotype^*a*^		Phenotype^*b*^		Cell Rounding^*c*^	Cytotoxicity^*c*^
*exoU*^+^	17	ExoU^+^	15	14/15	14/15
*exoT*^+^		ExoT^+^			
*popD*^+^		PopD^+^			
*pcrV*^+^		PcrV^+^			
		
		ExoU^-^	2	0/2	0/2
		ExoT^-^			
		PopD^-^			
		PcrV^-^			

*exoU*^-^	55	ExoU^-^	46	44/46	0/31
*exoT*^+^		ExoT^+^			
*popD*^+^		PopD^+^			
*pcrV*^+^		PcrV^+^			
		
		ExoU^-^	7	2/7	N.D.
		ExoT^-^			
		PopD^-^			
		PcrV^-^			
		
		other^*d*^	2	1/2	N.D.

*exoU*^-^	2	ExoU^-^	2	0/2	N.D.
*exoT*^-^		ExoT^-^			
*popD*^-^		PopD^-^			
*pcrV*^-^		PcrV^-^			

^*a *^Presence or absence of genes determined by PCR.

^*b *^Determined by ELISA.

^*c *^Results expressed as number of positive isolates/number of isolates tested.

^*d *^One isolate each with phenotypes ExoU^-^/ExoT^-^/PopD^+^/PcrV^- ^and ExoU^-^/ExoT^+^/PopD^+^/PcrV^- ^N.D., not determined.

We also assayed strains for secretion of T3SS proteins by immunoblotting using our polyclonal rabbit antiserum, which recognizes ExoU, ExoT, ExoS, PopB and PopD. This antiserum does not recognize the fourth known *P. aeruginosa *effector, ExoY, whose secretion does not appear to be associated with increased virulence in vitro or in vivo [25,26,34]. We found that the majority of clinical isolates secreted the effectors ExoU plus ExoT (n = 15) or ExoS plus ExoT (n = 45). Only one isolate secreted all three effector proteins (CI052), and only one other secreted ExoT alone. These findings are in agreement with other studies, which report that ExoU and ExoS are very rarely secreted by the same isolate [6,24,26] (data not shown). We were particularly interested in whether any of the 12 strains which scored negative for Type III protein secretion by ELISA would show detectable secretion of T3SS proteins by immunoblotting, especially as the maximum amount of supernatant assayed by Western (675 μl/lane, concentrated by TCA precipitation) was substantially greater than that assayed in our ELISA (5 μl/well). In only one case (CI051) were we able to detect secretion by immunoblotting that was undetectable by ELISA (data not shown). In all other cases, ELISA and immunoblotting assays yielded concordant results.

Many *P. aeruginosa *strains possess genes encoding the T3SS effectors, translocators and apparatus proteins, but nonetheless fail to produce or secrete these proteins [4,5,12,26]. We examined which clinical strains possessed the *exoU*, *exoT*, *pcrV*, and *popD *genes by PCR. We found that 12 strains did not secrete T3SS components as measured by ELISA even though they possessed the corresponding structural genes. We confirmed that our negative ELISA results were not due to extracellular protease production by these isolates by comparing assay results for a subset of these isolates (and the laboratory strain PA01) cultured in MinS or in MinS plus protease inhibitors (Complete™, EDTA-free, Boehringer-Mannheim). The presence of protease inhibitors completely inhibited the appearance of ExoT degradation products when culture supernatants were assayed by Western blotting with monoclonals TA and TB; however, no difference in ELISA results was noted for any isolate (data not shown). Thus, our failure to detect T3SS proteins was not due to their degradation by secreted proteases. We conclude, therefore, that T3SS secretion profiles are not accurately predicted by the genotype of a given strain in 16% of our clinical isolates.

Strains that do not secrete T3SS effectors may fail to synthesize these proteins or may fail to export them into the culture medium. We therefore further tested all strains that scored negative for T3S by ELISA and by immunoblotting, to see whether effectors are produced but retained within the bacteria. Bacteria were grown in MinS as per routine for ELISA assay, collected by centrifugation, and lysed with 4% SDS. The presence of T3SS components was assessed by immunoblotting as described in the Methods. We found that only one isolate (CI042) produced T3SS proteins but failed to secrete them into the culture medium (data not shown). Thus the ELISA and immunoblotting results were concordant for all clinical isolates with the exception of CI051, and only one isolate (CI042) produced but did not secrete TTSS proteins when grown in calcium-free medium.

### Secretion of TTSS proteins by *P. aeruginosa *cultured in calcium-free medium predicts TTSS-dependent virulence in cell-based assays

T3S is normally activated upon contact with host cells. However, T3S phenotypes are usually determined by culturing bacteria in media containing calcium chelators [4,5,12,26]. T3SS profiles of laboratory isolates determined in vitro usually predict ExoU-, ExoS- or ExoT-dependent effects on infected host cells. We examined whether this correlation held true for our clinical isolates. We thus assayed whether our clinical isolates cause rapid cytotoxicity, a phenomenon attributable to ExoU [8,11], and/or cell rounding and detachment, phenotypes associated with ExoT and ExoS translocation [35-37]. We included clinical isolates that were T3SS-negative by our ELISA to test whether they would elicit T3SS-dependent phenotypes upon contact with epithelial cells.

We first determined which strains caused rapid cytotoxicity by measuring LDH release from infected HeLa cells 2 h post-infection. We found that ExoU secretion in vitro as assayed by ELISA was strongly associated with the ability to cause tissue culture cell necrosis (see Table 4). 14 out of 15 isolates that secreted ExoU in vitro caused HeLa cell necrosis at 2 h post-infection, as measured by LDH release ≥25% of that seen with PA103 infected cells. One isolate, CI022, did not cause cell necrosis despite secreting ExoU in vitro. Interestingly, this isolate also did not cause cell rounding (see below), indicating that it fails to establish a productive interaction with epithelial cells. This strain is deficient for Type IV pilus-dependent motility, as evidenced by its lack of twitching motility in a subsurface agar stab assay [38] (data not shown). It is well-established that twitching motility deficient strains are markedly attenuated for T3SS-dependent virulence as measured by cell-based assays such as LDH release and cell rounding [39]. In agreement with ELISA results, no cell necrosis was caused by isolates CI034 and CI070, which possess the *exoU *gene but do not secrete ExoU in vitro. We attempted to correlate the amount of ExoU secreted by clinical isolates during growth in MinS with the amount of cytotoxicity these strains caused, as measured by LDH release at 2 hpi; however, no such correspondence was observed (data not shown).

We also performed cytotoxicity testing for 31 of the 45 isolates which were ExoU^-^/ExoS^+^/ExoT^+ ^and did not possess the *exoU *gene. None of the 31 isolates scored positive for LDH release (range, 0.2 to 3.2% of PA103 infected control at 2 hpi). This group included isolates that gave detectable ELISA values (5–8%) that were nonetheless below our 10% cut-off. Thus, the association between ExoU secretion as scored by ELISA and rapid cytotoxicity caused by our clinical isolates was robust.

We then performed cell rounding assays with each of the 72 clinical isolates that are positive by PCR for the minimum T3SS gene set of *pcrV*, *popD *and *exoT *(Table 4). As cell rounding is considered a measure of ExoT and/or ExoS translocation into host cells and subsequent disruption of the actin cytoskeleton [20,40], this assay serves as a sensitive test of whether the T3SS phenotype predicted by our in vitro ELISA test correlates with the capacity of each isolate to produce, secrete and translocate ExoT and/or ExoS upon contact with tissue culture cells. Bacterial strains lacking a functional T3SS (mutN) or lacking the effectors ExoU, ExoT and ExoS (PA103ΔUΔT) served as negative controls for this assay. All ExoU-secreting strains, with the exception of CI022 (see above), caused visible cell rounding in addition to measurable LDH release. All of these strains also secrete ExoT. Of the 47 ExoU-negative, ExoT-positive isolates assayed for cell rounding, 45 of 47 caused rounding equivalent to PAO1 (Table 4). The two isolates which failed to cause cell rounding, CI024 and CI047, do not exhibit twitching motility in a subsurface agar stab assay (data not shown).

We also assayed all 10 of the strains that were negative for protein secretion by ELISA, but possessed genes for *exoT*, *popD *and *pcrV*. Eight of these 10 isolates caused no cell rounding, as expected from the ELISA results. One of the two isolates that caused cell rounding was CI051, already mentioned above. ELISA and immunoblot results demonstrate that T3SS secretion by CI051 is approximately 5% that of the PA103 strain, which is below the cutoff for a positive ELISA result. The second isolate was CI042, which showed production, but not secretion, of T3SS components when grown in MinS. This suggests that CI042 might secrete T3SS proteins only in response to host cell contact, and not in response to MinS.

## Discussion

### ELISA-based detection of TTSS components of *Pseudomonas aeruginosa*

In this work we describe a rapid ELISA-based assay that detects secretion of four T3SS effector and translocator proteins. This assay shows excellent concordance with immunoblotting: only one sample classified as T3SS-negative by ELISA (CI051) showed T3SS protein secretion by immunoblotting, under conditions which allowed us to assay ca. 135-fold more antigen. The assay is easily adapted to clinical and research laboratory settings. The ELISA can analyze many samples simultaneously, at less cost and in less time than immunoblotting. In addition, the ELISA assay yields quantitative results, while immunoblotting typically gives qualitative results unless special procedures are followed. Thus, this assay is well-suited for clinical and research laboratory analysis of Type III secretion phenotypes of clinical and environmental *P. aeruginosa *isolates.

### Clinical isolates exhibit various deficiencies in their Type III secretion system

Several studies have found that *P. aeruginosa *clinical isolates show significant variability in secreting T3SS effectors [4,5,12,26]. Our study is no exception: a number of distinct secretion patterns appeared amongst the 74 isolates we examined. Sixty-three strains secreted a core group of effectors and translocators, namely ExoT, PcrV, PopD and PopB (the last assayed by immunoblotting). Fifteen of these strains also secreted ExoU, 46 strains secreted ExoS, one strain secreted both ExoU and ExoS, and one strain secreted neither. The presence of genes encoding these proteins in a *P. aeruginosa *isolate is not predictive of T3SS production or secretion. Seventy-two of 74 isolates possessed genes encoding PcrV, PopB, PopD and ExoT, but 11 failed to secrete any of these proteins as measured by ELISA and immunoblotting. Thus, methods based solely on detection of gene sequences in clinical isolates are unlikely to predict T3SS-dependent phenotypes. The absence of secretion was not generally due to defects in the secretion apparatus: only one clinical isolate (CI042) appropriately produced T3SS proteins but failed to secrete them into culture medium. The remaining 9 secretion negative isolates failed to produce T3SS proteins in response to growth in MinS, suggesting that the regulatory networks that induce transcription of T3SS genes in response to calcium chelation no longer function in these strains.

### T3S profiles in vitro predict T3SS-dependent virulence toward epithelial cells

The T3SS is triggered by contact between bacteria and host cells during infection, resulting in the translocation of bacterial effector proteins into host cells. We characterized all 74 clinical isolates in tissue culture cell-based assays which measure rapid cell necrosis or cell rounding and detachment, phenotypes which require the translocation of ExoU or ExoS/ExoT, respectively, into infected host cells. We then examined whether these phenotypes were predicted by the in vitro ELISA results we obtained for each clinical isolate. Sixty-nine of 74 isolates had phenotypes in tissue culture cell-based assays which were predicted by their T3SS profiles as measured by ELISA. Only one strain, CI051, caused cell rounding despite being scored as negative for T3SS secretion by ELISA. This isolate secreted very small amounts of T3SS proteins in vitro, which could only be detected by immunoblotting. Therefore, our cutoff value for classifying a strain as T3SS-positive versus negative, i.e. ≥10% of PA103 secretion levels, appears to be appropriate.

The remaining four strains whose phenotypes were not correctly predicted by our ELISA assay illustrate additional factors which influence bacterial virulence. CI022, CI024 and CI047, secreted ExoT, PopD and PcrV (as measured by ELISA), PopB (as demonstrated by immunoblotting), and ExoU (in the case of CI022), yet caused neither cell necrosis (CI022) nor cell rounding when used to infect HeLa cells. These strains are defective for Type IV twitching motility, which likely explains their apparent lack of virulence toward tissue culture cells [39]. Of note, strains deficient in twitching motility do show virulence in murine models of acute pneumonia [41](Kazmierczak, B.I., M. B. Lebron, T. S. Murray, submitted for publication). It still remains to be determined whether the absence of twitching motility in T3SS-positive strains influences the clinical outcomes observed during human infection.

The last isolate, CI042, caused cell-rounding but did not secrete T3SS proteins detectable by either ELISA or immunoblotting. We demonstrated that CI042 appropriately synthesized T3SS proteins, which could be detected by immunoblotting lysates prepared from bacteria cultured in either calcium-free medium or in the presence of host cells. Because HeLa cell rounding requires translocation of bacterial proteins across eukaryotic cell membranes, CI042 appears to exhibit the previously undescribed phenotype of selectively secreting T3SS effectors in response to host cell contact, but not to calcium chelation. Although it is possible that CI042 caused HeLa cell rounding and detachment through a mechanism independent of T3SS proteins, such as secretion of extracellular proteases, the following observations support a T3SS-based mechanism. When co-cultured with HeLa cells, CI042 caused cell rounding with the same kinetics as T3SS-positive strains and produced T3SS effectors that were detected in bacterial lysates prepared from infected HeLa cell cultures. Cell rounding activity could not be transferred in cleared and filtered supernatants prepared from CI042-infected cell cultures, suggesting that cell rounding activity was cell-associated and not secreted (L. L. and B. K., unpublished results). Thus, our results suggest that contact with epithelial cells may trigger secretion of TTSS effectors through a mechanism that is distinct from that used to respond to calcium depletion.

Thus in 69 isolates (93%), detection of secreted protein by ELISA correlated with phenotypic assays examining translocation of ExoU (cell necrosis) or of ExoT/ExoS (cell rounding and detachment). This indicates that secretion of T3SS effectors in vitro is an excellent predictor of T3SS effector translocation upon host cell contact for *P. aeruginosa *strains in general, including clinical isolates. Conversely, strains failing to express T3SS proteins under in vitro conditions are not likely to express them under conditions of host cell contact. The ELISA assay therefore represents a robust and easy to use tool for future studies correlating in vitro behavior of *P. aeruginosa *clinical isolates with in vivo outcomes of infection.

### Clinical significance of TTSS-negative clinical isolates

The ability of isogenic series of *P. aeruginosa *mutants constructed in the laboratory to cause disease in murine models of pneumonia is strongly correlated with their ability to secrete and translocate the effectors ExoU and, to a lesser extent, ExoS [24,25,34]. Several studies examining T3SS profiles of clinical *P. aeruginosa *isolates isolated from human patients concluded that T3SS-positive strains were associated with more severe clinical disease [4-6,26]. In each study, however, T3SS-negative strains were also isolated from patients who appeared to have severe *P. aeruginosa *infections. No study was designed to control for host factors that might have influenced severity of infection, such as underlying host disease or immunocompromise. As each study relied on a single banked clinical specimen per patient, no study could address the possibility that more than one *P. aeruginosa *strain was present during infection. Jain *et al*. recently observed that cystic fibrosis patients can be colonized by multiple strains that show heterogeneity in their T3SS protein secretion profiles, despite being genotypically indistinguishable. It remains to be determined whether heterogeneous isolates are also present in acutely infected patients. Virulence factors other than T3SS proteins may also contribute to clinical virulence of *P. aeruginosa *strains. The presence or absence of surface structures, such as Type IV pili and flagella, has been correlated with changes in virulence in animal models of infection [42-44]. To our knowledge, however, no study has systematically examined the influence of such structures on disease severity caused by T3SS^+ ^or T3SS^- ^strains.

In conclusion, the ability to carry out a rapid, simple and inexpensive assay to determine T3SS profiles enables prospective studies investigating the relationship between *P. aeruginosa *expression of these virulence factors and clinical manifestations of infection in a large patient cohort. Phenotypic assays, including TTSS effector production, may ultimately have significant prognostic value for clinicians caring for patients from whom *P. aeruginosa *is cultured.

## List of abbrevations used

Type III secretion system, T3SS; enzyme-linked immunosorbent assay, ELISA; nitrilotriacetic acid, NTA; Luria Broth, LB; paranitrophenyl phosphate, PNPP; phosphate buffered saline, PBS; trichloroacetic acid, TCA; horseradish peroxidase, HRP; lactate dehydrogenase, LDH.

## Authors' contributions

ML purified protein antigens, optimized ELISA format, carried out ELISA analyses of clinical isolates, and participated in drafting and revising the manuscript. LL generated and screened all hybridoms, maintained clinical strain collection, carried out all phenotypic assays and immunoblotting experiments, and participated in manuscript preparation. KK participated in screening hybridomas and ELISA analyses of clinical isolates. RAK participated in manuscript preparation. BIK conceived of the study, carried out animal immunizations, had primary responsibility for manuscript preparation and revision. All authors were involved in experimental design and analysis of data. All authors read and approved the final manuscript.
